# Pyorrhœa Alveolaris

**Published:** 1899-07

**Authors:** Harry F. White

**Affiliations:** University of Pennsylvania


### PYORRHŒA ALVEOLARIS.

BY HARRY F. WHITE, UNIVERSITY OF PENNSYLVANIA.

RECORD of a case which presented itself to me in the clinic during session of 1898–99.

*Symptoms.*—This person was a woman of about thirty-five years. She wore an upper denture, the only natural teeth remaining being the ten lower anterior teeth. She complained of the gums feeling uncomfortable, and of the teeth being loose, especially the second bicuspid, on left side, and the two central incisors, these teeth being most affected.

The gums were somewhat swollen and inflamed and of a rather dark-purplish color. Pressure along gingival margins caused pus to flow from necks of those teeth most affected. The gums were also receded.

*Diagnosis.*—My diagnosis was based on the following facts: (*a*) Inflamed gums; (*b*) pus flowing from necks; (*c*) loosened teeth; (*d*) deposits of tartar on roots; (*e*) recession of gums.

*Cause.*—The disease was undoubtedly caused by over-use of the few remaining teeth. This extra strain set up irritation of pericementum and gingivitis, making a culture-pocket for bacteria.

*Prognosis.*—Without treatment these teeth would have continued to loosen till all were lost, but the prognosis was very favorable with the exception of one bicuspid, which was very loose.

*Treatment.*—1. First treatment was to thoroughly clean and polish roots of all teeth and to cleanse pockets with hydrogen dioxide.

2. The next was to syringe pockets with warm water and apply a twenty-five-per-cent. solution of sulphuric acid, carried in pockets with cotton on an orange-wood stick. This was left in from three to five minutes, after which bicarbonate of soda was applied to neutralize acid. I then syringed with warm water and packed in sulphate of quinine.

3. A wash was then given consisting of

℞ Hydronaphthol, gr. xv;

Alcoholis,

Aquæ destillatæ, āā ℥i. M.

Sig.—Twenty drops in glass of water, to be used twice daily.

At each succeeding sitting I saw great improvement. The pockets were cleansed again with warm water and more quinine packed in. This treatment continued till all the teeth were treated. The patient was seen each day, and three teeth were treated at each sitting.

Have seen patient several times since. The gums are now in perfectly healthy condition. The teeth have tightened in their sockets and appear to be normal.

---

